# Prescribing patterns for hyperopia: an insight of the optometrist perspective and practice

**DOI:** 10.1186/s12886-024-03496-5

**Published:** 2024-05-28

**Authors:** Ali Alsaqr, Saleh Alhumaid, Muteb Alanazi, Ali Abusharha

**Affiliations:** Department of Optometry, College of Applied Medical Sciences, King Saud University, P.O. Box 10219, 11433 Riyadh, Saudi Arabia

**Keywords:** Hyperopia, Hyperopia management, Optometry, Clinical practice, Saudi Arabia

## Abstract

**Background:**

To investigate the current prescribing patterns for correcting hyperopia among optometrists in clinical practice in Saudi Arabia and compare those to current international guidelines. And explore the factors that influence practitioners' prescribing decision.

**Method:**

This cross-sectional study employed 30 items online survey that encompass demographic data, current practice and cycloplegia use, numerical response to indicate the minimum level of hyperopia at which optometrists would consider prescribing spectacles to non-strabismic children and determine the diopter value required for prescribing correction for hyperopia if present with other factors.

**Result:**

A total of 104 optometrists responded to the survey (52 females and 52 males). They recruited from 35 cities across Saudi Arabia. Out of total, 44% of them considered cycloplegic refraction essential under 12 years and 56% of them extended the range to 18 years. Large variation were found between the optometrists’ responses and current guideline recommendations. Several factors influenced the decision-making of the practicing optometrist including signs and symptoms, bilateral hyperopia, average dioptric value, reading difficulty, and accommodative function.

**Conclusion:**

There are some matches between the international guidelines and the practice patterns that followed by optometrists in Saudi Arabia, however, the optometrists did not report that they are following them purposefully. These findings highlight the need to improve optometrists' practice about spectacle prescription in pediatric population.

**Supplementary Information:**

The online version contains supplementary material available at 10.1186/s12886-024-03496-5.

## Background

One of the most common causes of vision impairment in children is refractive error [1]. The refractive errors affect around 116 million individuals worldwide and account for 53% of visual impairment [2]. Further, uncorrected refractive errors are the major cause of moderate to severe vision impairment and the second leading cause of blindness worldwide, according to a recent World Health Organization report [2]. Uncorrected refractive error can cause non-reversible eye conditions, including amblyopia and strabismus. A successful management of refractive errors not only reduces the risk of developing these problems, but also provides feasibility for binocularity and stereopsis to develop normally [1].

Specifically, refractive errors are the most common cause of amblyopia in Saudi Arabia [3]. Uncorrected refractive error has an immediate and long-term influence on an individual's education, profession, and quality of life [4]. As a result, proper refractive error management is crucial for preventing amblyopia and ensuring appropriate binocularity and stereopsis development. Further, Myopia, hyperopia, and astigmatism are reported to be prevalent in 11.7%, 4.6%, and 14.9% of children worldwide [5]. While in Saudi Arabia, uncorrected refractive errors were estimated to be 34.9% in Medina [6], 22% in Jazan [4], whereas the general prevalence of refractive errors was 13.7% in Alahassa [7],18.6% in Alqassim [8], and 13% in Riyadh [9]. The most typical and cost-effective method of correcting refractive errors in children is spectacle correction [1].

Hyperopia is a common refractive error that affects both children and adults [10]. Hyperopia occurrences can be accounted for greatly physiological causes (i.e., axial length, flat corneal curvature, crystalline lens power) [10]. Hyperopia is also linked by hereditary factors, with the environment playing a role in the development and severity of the problem [10]. Symptoms of hyperopia vary depending on the degree of hyperopia, the individual's age, the state of accommodation and convergence, as well as the demands placed on the visual system (distance vs. near) [10]. Some young hyperopia patients, particularly those with moderate and high hyperopia, may have few signs and symptoms [10]. The signs and symptoms of hyperopia could involve red or tearing eyes, squinting and facial contortions while reading, ocular fatigue or asthenopia, frequent blinking, constant or intermittent blurred vision, focusing problems, decreased binocularity and eye–hand coordination, and difficulty or aversion to reading. The presence of these symptoms, as well as their intensity, may varies greatly. Therefore, early detection of hyperopia may help in preventing the occurrence of strabismus and amblyopia in young children. Uncorrected hyperopia can impair learning capacities in young children, and it can also cause ocular discomfort and visual inefficiency in people of all ages [10].

Atkinson and colleagues suggested that uncorrected hyperopia (> 3.5 diopters (D) in one meridian) has been linked to impaired motor and cognitive development in young children, aged 9 months to 5.5 years, and learning issues in certain older children [11]. Although the exact mechanism of this association remain unknown, optical blur, accommodative and binocular dysfunction, and fatigue all appears to play a significant role [11]. Uncorrected infant hyperopia has been linked to moderate delays in visuo-cognitive and visuo-motor development but seems to achieve level of their emmetropic counterparts following 6 weeks of full-time hyperopic spectacle use in 3–5-year-olds [11]. The large percentage of school-age children with uncorrected high hyperopia demonstrated potential effect on learning-related skills and showed the importance of screening programs in early detection of refractive errors [10]. According to the American Optometric Association [12], hyperopia can be classified into (1) low hyperopia <  + 2.00 D; (2) moderate hyperopia of between + 2.25 and + 5.00 D; and high hyperopia of >  + 5.00 D.

The emmetropization process leads to gradual decrease in the level of hyperopia in most individuals [13]. Children with high hyperopia are more likely to remain hyperopic throughout childhood [13]. In comparison to hyperopic newborns without considerable astigmatism, children with high hyperopia may have a higher incidence of against the rule astigmatism, which appears to reduce the decline in hyperopia during emmetropization period [13]. Hyperopia greater than + 3.25 D affects up to 9% of 6 to 9 months old newborns, and this percentage drops to 3.6% at one year old population [13].

The hyperopic spectacle correction shall be tailored to the specific needs for every child. Some of the factors that can be considered when consider management options would involve the patient's age, the amount of hyperopia (under dry and cycloplegic refraction), amount of astigmatism, anisometropia, esotropia, amblyopia, the state of the accommodative and convergence, the demands imposed on the visual system and any symptoms [10]. Corrective spectacles are considered a cost-effective intervention [14]. However, pediatric spectacle prescription in preschool children is difficult due to several reasons. These would include, for example but not limited to difficulty assessing children due to lack of cooperation and varying practitioner's judgments for the same refractive defect. Furthermore, children's visual system face unique hurdles, rendering them more vulnerable to amblyopia caused by refractive errors [15].

Correction for hyperopia in asymptomatic patient is generally depends on the amount of hyperopia in relation to the patient's age, as well as preferred practice patterns [16]. There have been a number of guidelines developed over the years to assist optometrists in prescribing for refractive errors in children (Table 1) [17]. The American Academy of Ophthalmology (AAO) released guidelines based on professional consensus [18], whereas Miller and Harvey proposed recommendations based on consensus among members of the American Association for Pediatric Ophthalmology and Strabismus (AAPOS) [19]. The guidelines for the Royal College of Ophthalmology (RCO) have been developed by various groups of practitioners, including pediatric ophthalmologists, orthoptists, ophthalmologists, and optometrists [20]. Finally, Susan Leat in 2011 develop prescription recommendations for various refractive disorders in children based on literature review and clinical opinion [17].

**Table 1:** Summary of guidelines for patients with hypermetropia

	< 1 years	1–2 years	2–4 years	4–7 years
AAO^[17]^ [18]	$\ge$+ 6.00	$\ge$+ 5.00	$\ge$+ 4.50	No specific numbers, prescribe based on symptoms
AAPOS^[18]^ [19]	$\ge$+ 4.50		$\ge$+ 4.00	$\ge$+ 3.50
RCO^[19]^ [20]	$\ge$+ 4.00			
Susan Leat^[16]^ [17]	$\ge$+ 3.50			$\ge$+ 2.50

^a^*AAO* American Academy of Ophthalmology, *AAPOS* American Association for Pediatric Ophthalmology and Strabismus, *RCO* Royal College of Ophthalmology

In Saudi Arabia, Farah and Zainab in 2020 surveyed practicing optometrists in Riyadh to evaluate the prescribing philosophies for asymptomatic hyperopic children [21]. They reported that they will prescribe correction for children up to 7 years if the presented hyperopia was > 3 diopters [21]. This study had small sample size, 26 optometrists, and they did not include factors that may influence their decisions including signs and symptoms. A second study was conducted to explore the approach to prescribing glasses and the interpretation of refractive errors in children among ophthalmologists/optometrists in Saudi Arabia [22]. They found significant differences between the participants’ responses and actual practices based on guideline suggestions. This study recruited 49 optometrists and had only 5 questions, based on case scenarios, regarding hyperopia prescription [22]. Both studies did not focus on exploring whether optometrists follows a specific guideline or not.

This study is trying to cover all aspect regarding hyperopia prescription in non-strabismic children and include factors that may influence practitioners' decisions when prescribing correction for refractive errors including signs and symptoms among optometrists in clinical practice in Saudi Arabia. It will also investigate the current prescribing patterns for correcting hyperopia and compare them to international guidelines.

## Materials and method

This study was a cross-sectional study in design. The target population were optometrist in Saudi Arabia. The sample size was computed using Epi Info, version 7.2 (Centers for Disease Control, USA; https://www.cdc.gov/epiinfo/pc.html). The entries were a population size of 1900 practitioners, with expected frequency of 75% (indicating that two third of them examine children), the design effect was set at 2, 95% confidence interval and the number of clusters were 5 clusters (central region, northern region, western region, eastern region and southern region). The returned estimated sample size was noted (*n* = 100). All Saudi Arabia qualified optometrists were targeted in this study.

The data were collected using an online self-administered survey in English (Google forms). The electronic link was sent to 600 practicing optometrists with an introduction of the study. A further reminder emails were sent to the optometrists two and four weeks after the first email was sent for those optometrists who did not respond in order to increase the percentage of response rate. The social media such as twitter and WhatsApp were also employed.

The survey was designed based on previously published questionnaires [23, 24]. In total, 30 questions were included in the survey (Appendix 1). The information gathered included respondent's demographic data, current practice and cycloplegia use. Also the respondents were asked to provide a numerical response in the form of a dioptric value to indicate the minimum level of hyperopia, myopia, anisometropia, and astigmatism at which they would consider prescribing spectacles to non-strabismic children aged 1, 3, 5, 7, 9, and 11 years. Finally, the respondents were asked to determine the diopteric value required for prescribing correction for hyperopia when present with other factors. Statistical Package for the Social Sciences (version 26; IBM Corp., Armonk, NY, USA) was used for all data analyses. Descriptive analyses were executed to determine the percentage of respondents for every item.

## Result

A total of 104 optometrists responded to the survey. The respondents were 52 females and 52 males, and their age was 30 ± 9 years, ranged from 24 to 57 years old. In Saudi Arabia only two colleges provide optometry degree, therefore, most of them graduated from King Saud University (n = 82, 79%), followed by 19% (n = 19) whom graduated from Qassim University. The rest of respondents graduated from other universities outside Saudi Arabia such as Al-Neelain University, Sudan; University of Manchester, UK. Bachelor's degree was the most prominent in this survey which account to 61% (n = 63), 29% (n = 30) of them have MSc degree and 10% (n = 11) have a PhD degree. The median years of experience was 5 years (ranged 1 to 28 years).

The central region had the most responses (64%, n = 67), followed by western region (13%, n = 14), northern region (10%, n = 10), eastern region (8%, n = 8), and southern region (5%, n = 5). The respondents were recruited from 35 cities across Saudi Arabia, and most of them were from Riyadh in central region (56%, n = 58), followed by Jeddah (8%, n = 8), then Buraidah (6%, n = 6), while the rest of respondents distributed evenly between the other cities. Further, the primary area of the respondents’ eye care activities was general eye care services (71%, n = 74), while 19% (n = 20) focused on providing podiatric service and the rest reported that they provide CL and other optometric service (10%, n = 10). Their organization of attachment were hospitals (75%, n = 78), multiple practices (7%, n = 8), independent practice (6%, n = 6), academic institution (5%, n = 5), primary eye care center (4%, n = 4) and optical center (3%, n = 3). Most of the respondents work as full time optometrist (91%, n = 93) and 11% (n = 11) as part timer. Finally, when the respondents were asked about number of children that they encounter per week, the distribution of responses varied in accordance of the children age, with more children are encountered in older age (Table 2).

**Table 2:** Number of children that optometrist examined every week in accordance with their age

No. children	< 10 children	10 – 20 children	> 20 children
Infants < 12 month	91%, n = 95	6%, n = 6	3%, n = 3
Infants 12—24 months	84%, n = 88	9%, n = 13	3%, n = 3
Foundation (2—4 years)	56%, n = 59	33%, n = 34	11%, n = 11
Key stage 1 (5—7 years)	26%, n = 27	53%, n = 55	21%, n = 22
Key stage 2 (8—11 years)	25%, n = 26	46%, n = 48	29%, n = 30

**Fig. 1:**
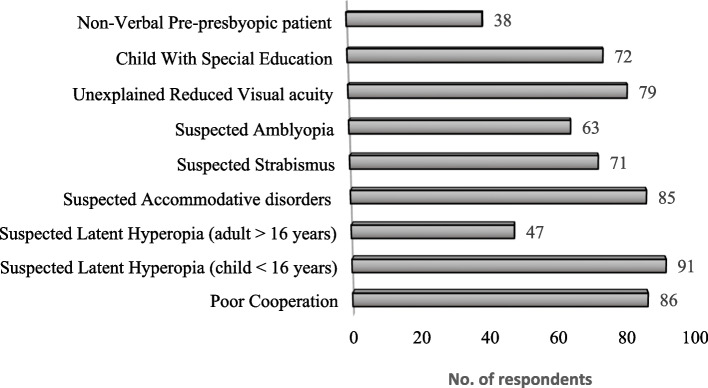
Main circumstances other than age for considering the use of cycloplegia reported by the respondents

In the second part of the survey, when asked about up to what age they consider cycloplegic refraction essential at the child's first visit, the median was up to10 years (ranged 1 to 18 years), 44% of the respondents reported considered the maximum age was 12 years while 56% of the respondents extended the range to 18 years. Additionally, when they were asked for additional circumstances other than the age for considering the use of cycloplegia, the most frequent responses were poor cooperation and suspected latent hyperopia (child < 16 years), alongside other factors that is summarized in Fig. 1.

**Fig. 2:**
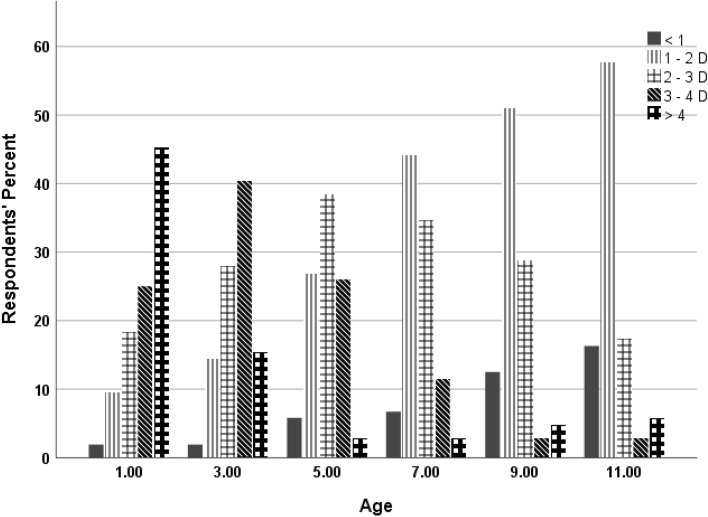
Showing the minimum dioptric values of hyperopia, that practitioners would consider prescribing in non-strabismic children at ages 1, 3, 5, 7, 9 and 11 years

The minimum levels of hyperopia at which the respondents would consider prescribing spectacles in children with non-strabismus at each specific age are shown in Fig. 2. Overall, the respondents are likely to have more tendency to prescribe for lower hyperopic power as the child gets older (Fig. 2). Briefly, 49% would prescribe > 4 D for children at year 1, 42% would prescribe 3–4 D for children at 3 years, 40% would prescribe 2–3 diopter for children at 5 years, 46% would prescribe 1–2 diopter for children at 7 years, 53% would prescribe 1–2 diopter for children at 9 years, and 60% would also prescribe 1–2 diopter for children at 11 years.

When respondents were asked about other factors influence their prescribing decision, about 92% of them would prescribe correction for hyperopia if present with symptoms and the least factor was if present with parent or child preferences (9%). Table 3 shows all factors influence whether respondents would prescribe for children with hyperopia.

**Table 3:** Percentages of practitioners who reported considering the listed factors and the diopteric value associated with these factors when prescribing for hyperopia

Factor	Respondents Whom considering factor at all when prescribing	% Who considering diopter value associated with factor				
		1 D	2 D	3 D	4 D	Not prescribe
Symptoms	92%	43%	47%	4%	4%	2%
Reading problems	85%	64%	30%	4%	0%	2%
Accommodative dysfunction	81%	64%	29%	6%	0%	1%
Esophoria	71%	58%	27%	11%	2%	2%
Presence of motor / neurodevelopment problems	25%	37%	23%	13%	7%	20%
Decrease near visual acuity	75%	60%	30%	7%	3%	0%
Decrease stereoacuity	35%	36%	36%	8%	3%	17%
Refractive error at prior eye exam	36%	41%	23%	16%	3%	17%
Family history	33%	20%	28%	17%	8%	27%
Parent or child preferences	9%	26%	25%	15%	1%	33%

**Fig. 3:**
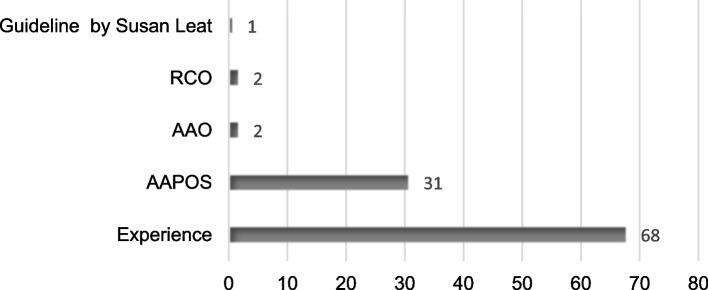
Show guidelines that the reported that they are following in their prescribing decision. RCO, Guideline of Royal College of Ophthalmologists; AAO, Guideline of American Academy of Ophthalmology; AAPOS, Guideline of American Association of Pediatric Ophthalmology and Strabismus

The participants were asked which guidelines they are following when prescribing correction for hyperopia and the most common response was following their experience and clinical opinion judgment (65%) followed by AAPOS guideline (30%) (Fig. 3).

### Comparing current findings with previous guideline and findings

In Table 4, a comparison summary of the cut-off dioptric values when prescribing for asymptomatic hyperopia founded in the current study and previous studies. There were some differences between this study and data reported in previous studies, and it was more marked with AAO guideline. Although, some of the main findings were similar to previous studies (Table 3). Specifically, Susan Leat guideline was more comparable to the current results in children up to 4 years old and slightly different in children between 4 and 7 years old (0.50 D), although the respondents did not report that they are following Susan Leat guideline.

**Table 4:** Shows mean diopteric values for prescribing hyperopia from current study and the listed previous guideline

	< 1 years	1–2 years	2–4 years	4–7 years	7–11 years
AAO [17]	≥ + 6.00	≥ + 5.00	≥ + 4.50	No specific numbers, prescribe based on symptoms	
AAPOS [18]	≥ + 4.50		≥ + 4.00	≥ + 3.50	NR
RCO [19]	≥ + 4.00				NR
Susan Leat [16]	≥ + 3.50			≥ + 2.50	NR
Doyle et al. [20]	> + 3.50		> + 2.50	> + 2.00	> + 1.50
Farah and Zainab [22]	> + 3.00				NR
Current study	Average				
	> + 3.50			> + 3.00	> + 2.50
	Majority				
	> + 4.00		> + 3.50	> + 2.50	> + 1.50

*AAO* guideline of American Academy of Ophthalmology, *AAPOS* American Association of Pediatric Ophthalmology and Strabismus, *RCO* guideline of Royal College of ophthalmology, *NR* not reported

## Discussion

Uncorrected hyperopia in non-strabismus children is a risk factor for amblyopia and have impact of child quality of life as well as their performance in school [25]. In Saudi Arabia, one study screened 5176 primary school children and reported that Mild-to-moderate hyperopia accounted for the majority of all hyperopic cases (89%) encountered [8]. This would emphases the importance of both knowledge and standards of how to manage hyperopia in young children. This cross-sectional study is of importance in that it does not focuses only on optometrists’ decision-making approach but provided in-depth insight about prescribing pattern for correcting hyperopia in non-strabismus children, factors influences their decision and cut-off dioptric power that would influences prescription of spectacle for children among optometrists in Saudi Arabia.

Several factors shall be considered when prescribing spectacles for hyperopia including child age, hyperopic power, amount of anisometropia, and presence of strabismus or amblyopia [1]. Emmentropization is another significant issue to consider. Many newborns were found to be hyperopic, with an average cycloplegic refractive error of + 2 diopters [26]. From the age of 3 to 12 months, there is a rapid emmetropization process [27]. The underlying refractive error, however, influences the emmetropization process. The possibilities of reaching full emmetropization decreases as initial refractive errors increase [28]. Emmetropization can last for up to 6 years in spherical ametropia and even longer in certain mild hyperopes [17]. Emmetropization should be allowed to develop naturally, with spectacles prescriptions assisting it. Prescribing spectacles are needed to move the visual system into a refractive tolerance envelope, after which emmetropization could takes control. Therefore, several guidelines have been developed to determine the amount of spectacles prescription required at each age.

Doyle and colleagues [23] study was conducted to find out what UK optometrists response about using cycloplegia and prescription spectacles to children up to 11 years old. In their study the focus was on using cycloplegia among optometrists and there was a fewer questions regarding refraction and spectacle prescription. Further, other factors including signs and symptoms that may influence practitioners' decisions while prescribing were not included. Similar to the finding of current study, they reported average dioptric value for prescribing correction for hyperopia in non-strabismus children were closely comparable to Susan Leat guideline [17]. In addition, our result matched the Doyle et al. study findings [23] in 1 to 2 years old children group and in children of 7 to 11 years old group. Further, Kulp and colleagues [24], contacted pediatric eye care practitioners to determine current prescribing practices for correcting hyperopia, considering the degree of hyperopia and other factors. They reported that, symptoms, presence of astigmatism and/or anisometropia, reading difficulty, and accommodative function were most considered when prescribing for hyperopia. They reported that the amount of hyperopia prescribed by more than half of all eye care practitioners declined with increasing child age. These findings are also in alignment with this study observations. Finally, in the present study, 36% of respondents reported that they were following AAPOS guideline. However, the average of dioptric value of the current result was closer to Susan Leat guideline, although they were not purposefully following it, and greatly different from AAPOS.

In general, the present study demonstrated some differences in prescribing pattern in comparison to those reported by AAO, AAPOS, RCO and other guidelines in Table 3. This finding was also consistent at the local level when compared to previous study in Riyadh city [21]. This may be due to sample size; the present study was three times larger than their study. And their study was done only in one city while present study was done in 35 cities. The second study conducted in Saudi Arabia, was investigating ophthalmologists and optometrists practicing in Saudi Arabia [22]. They recruited 49 optometrists and had 10 questions and 5 of them were case scenarios about hyperopia prescription. Although there was difference in methodology between the current study and their study, some agreement was found. For example, in one scenario concerning the management of moderate hyperopia, 10% would prescribe according to refraction which matches with current study; where most of participants of current study would not prescribe + 1.50 D hyperopia for 3 years olds children. There earlier two studies did not explore whether optometrists follows a specific guideline or investigate factors impacting practitioner decision.

In order to acquire an "accurate refraction," the RCO recommends cycloplegic refraction in children under the age of 12 [29]. The result in this study showed that 44% of the optometrists consider cycloplegic refraction is essential under 12 years and 56% of them extended the range to 18 years. This may indicate that the optometrists is precautious in managing hyperopia.

When considering factors influence decision-making regarding prescribing spectacles for hyperopia, optometrists in current study differed greatly for every factor except symptoms and reading problems. Of them, 93% of participants will consider symptoms if present with 2 diopter and this was comparable to Kulp et. al. study (98% will consider symptoms) and Cotter's recommendation in USA (prescribing + 1.50 D if they associated with symptoms or reading problems) [13, 24]. In addition, 85% of participants in this study was considering reading problems and the majority was consider if present with one diopter or more and this almost compatible with Cotter's recommendation that mention earlier [13]. These was less than the result reported in Kulp et. al. study [24], they report that 99% of optometrist will consider reading problems when prescribing correction for hyperopia. Among factors showing the least considering in present study when prescribing correction was presence of and family history (33%). This factor is of crucial necessity and more education and awareness might be needed.

Visual impairments are more common in children with neurodevelopmental disorders, according to the RCO and the UK government's National Service Framework for Disabled Children [30, 31], these children should have a routine vision check as part of their multidisciplinary treatment. For example, children with Down syndrome had less accommodation ability [32]. However, current study showed that only 25% of participants will consider neurodevelopment disorder when prescribe correction for hyperopia. Which may indicate that more attention shall be directed at this significant factor.

This study had some limitations, firstly, online surveys are effective in mass distribution nonetheless have their challenges including small sample size and poor response rate. Further, multiple-choice questions may not provide the optometrist the chance to reflect on day to day practice patterns, but they are the effective approach to explore the variance in decision-making between optometrists. However, this study is one of few attempts to describe the spectacle prescribing pattern in the young children among optometrists in Saudi Arabia. This study findings can assist in identifying practice gaps that can help optometrists when prescribing spectacles for hyperopia in young patients.

In conclusion, practice patterns regarding children spectacle prescription varied among optometrists in Saudi Arabia. Although there are some matches between some of the international guidelines and the current pattern that followed by optometrists in Saudi Arabia, optometrists didn’t report that they are following any of them. These findings highlight the need to improve optometrists' awareness regarding the prescription of spectacles in the children population. Future studies can be directed to develop national guidelines to help optometrists when prescribing spectacles in children with hyperopia.

### Supplementary Information

Supplementary Material 1.

## Data Availability

All the relevant data have been provided in the manuscript. Supplementary datasets used and/or analyzed during the current study are available from the corresponding author upon reasonable request.

## References

[1] Sharma P, Gaur N (2018). How do we tackle a child's spectacle?. Indian J Ophthalmol.

[2] Flaxman SR (2017). Global causes of blindness and distance vision impairment 1990–2020: a systematic review and meta-analysis. Lancet Glob Health.

[3] Aldebasi YH (2015). Prevalence of amblyopia in primary school children in Qassim province, Kingdom of Saudi Arabia. Middle East Afr J Ophthalmol.

[4] Al Bahhawi T (2018). Refractive error among male primary school students in Jazan, Saudi Arabia: prevalence and associated factors. Open Ophthalmol J.

[5] Hashemi H (2018). Global and regional estimates of prevalence of refractive errors: Systematic review and meta-analysis. J Curr Ophthalmol.

[6] Alrahili NHR (2017). Prevalence of uncorrected refractive errors among children aged 3–10 years in western Saudi Arabia. Saudi Med J.

[7] Al Wadaani F (2013). Prevalence and pattern of refractive errors among primary school children in Al Hassa, Saudi Arabia. Glob J Health Sci.

[8] Aldebasi YH (2014). Prevalence of correctable visual impairment in primary school children in Qassim Province, Saudi Arabia. J Optom.

[9] Alsaqr AM (2017). Investigating the visual status of preschool children in Riyadh, Saudi Arabia. Middle East Afr J Ophthalmol.

[10] Dartt DA (2010). Encyclopedia of the Eye.

[11] Atkinson J (2005). Refractive errors in infancy predict reduced performance on the movement assessment battery for children at 3 1/2 and 5 1/2 years. Dev Med Child Neurol.

[12] Amos JF. Diagnosis and management in vision care. UK: Butterworths, Ltd; 1987.

[13] Cotter SA (2007). Management of childhood hyperopia: a pediatric optometrist's perspective. Optom Vis Sci.

[14] Padhye AS (2009). Prevalence of uncorrected refractive error and other eye problems among urban and rural school children. Middle East Afr J Ophthalmol.

[15] Donahue SP (2007). Prescribing spectacles in children: a pediatric ophthalmologist's approach. Optom Vis Sci.

[16] Lyons SA (2004). A survey of clinical prescribing philosophies for hyperopia. Optom Vis Sci.

[17] Leat SJ (2011). To prescribe or not to prescribe? Guidelines for spectacle prescribing in infants and children. Clin Exp Optom.

[18] Cruz OA. et al. Pediatric ophthalmology/strabismus preferred practice pattern development process and participants. 2022 [cited 2022 October]; Available from: https://www.aao.org/education/preferred-practice-pattern/amblyopia-ppp-2022.

[19] Miller JM, Harvey EM (1998). Spectacle prescribing recommendations of AAPOS members. J Pediatr Ophthalmol Strabismus.

[20] Royal College of Ophthalmologists. Guidelines for the management of amblyopia. 2010 [cited 2022 September]; Available from: http://www.rcophth.ac.uk/docs/publications/GuidelinesfortheManagementofAmblyopia.pdf

[21] Maqsood F, Alhawas ZA (2020). Clinical prescribing philosophies survey for hyperopia in Riyadh. J Clin Diagnost Res.

[22] Malaika R (2020). Pediatric spectacle prescription: Understanding practice patterns among ophthalmologists and optometrists in Saudi Arabia. Saudi J Ophthalmol.

[23] Doyle LA, McCullough SJ, Saunders KJ (2019). Cycloplegia and spectacle prescribing in children: attitudes of UK optometrists. Ophthalmic Physiol Opt.

[24] Kulp MT (2021). Prescribing Patterns for Hyperopia. Invest Ophthalmol Vis Sci.

[25] Castagno VD (2014). Hyperopia: a meta-analysis of prevalence and a review of associated factors among school-aged children. BMC Ophthalmol.

[26] Gwiazda, JE. et al. Emmetropization and the progression of manifest refraction in children followed from infancy to puberty. Clin vis scie. 1993;8:337–44.

[27] Mayer D (2001). Cycloplegic refractions in healthy children aged 1 through 48 months. Arch Ophthalmol.

[28] Mutti DO (2009). Accommodation, acuity, and their relationship to emmetropization in infants. Optom Vis Sci.

[29] The Royal College of Ophthalmologists. Guidelines for the Management of Strabismus in Childhood. 2012 [cited 2022 September]; Available from: https://www.rcophth.ac.uk/resources-listing/guidelines-for-the-management-of-strabismus-in-childhood/.

[30] Ophthalmologists, T.R.C.o. Ophthalmic Services for Children. 2012. [cited 2022 September]; Available from: https://www.rcophth.ac.uk/resources-listing/ophthalmic-services-for-children/.

[31] Sloper P, Statham J (2004). The National Service Framework for children, young people and maternity services: developing the evidence base. Child Care Health Dev.

[32] Little J-A (2015). Accommodation deficit in children with Down syndrome: practical considerations for the optometrist. Clin Optom.

