# Supplementary material for: Prescribing patterns for hyperopia: an insight of the optometrist perspective and practice
Source: BMC Ophthalmol. 2024 May 28;24:226. doi: 10.1186/s12886-024-03496-5 (PMC11134738; doi:10.1186/s12886-024-03496-5)
Supplement: Supplementary file 1 — Supplementary Material 1. [file 12886_2024_3496_MOESM1_ESM.docx]

Appendix

[1]. Questionnaire that uses in this study:

1. Gender

- Male
- Female

1. Age (years)

………………..

1. Qualification

- BSc.
- MSc
- PhD
- Fellowship pediatric ophthalmology
- Other……………

1. At which institution did you complete your undergraduate degree?

………………………………

1. How many years have you been in-practice (in approximate years)?

………………………..

1. Organization of attachment

- Multiple
- Independent
- Hospital
- Academia
- Primary care center
- Other (please specify)…..

1. Practice location (region)

- Northern Region
- Southern Region
- Eastern Region
- Western Region
- Central Region

1. Practice location (city)

…………………………

1. Primary areas of your eye care activity

- General eye care
- Pediatric eye care services
- Other …………

1. Practice status

- Full time
- Part time

1. Approximately how many children in the following age groups do you see each week?

|  | Less than 10 | 10-20 | More than 20 |
| --- | --- | --- | --- |
| Infants <12 months |  |  |  |
| Infants 12 - 24 months |  |  |  |
| Foundation (aged 2 - 4 year) |  |  |  |
| Key Stage 1 (aged 5 - 7 years) |  |  |  |
| Key Stage 2 (aged 8 - 11 years) |  |  |  |

1. Up to what age (in approximate years) do you consider cycloplegic refraction ESSENTIAL at a child's FIRST VISIT?

………………………

1. Other than age, in what additional circumstances would you consider the use of cycloplegia? (multiple selection you may choose more than one choice)

- Poor Cooperation
- Suspected Latent Hyperopia (Child <16 years)
- Suspected Latent Hyperopia (Pre-presbyopic Adult >16 years)
- Suspected Accommodative Disorder
- Suspected Strabismus
- Suspected Ambylopia
- Unexplained Reduced Visual Acuity
- Child With Special Educational Needs
- Non-Verbal Pre-presbyopic Patient
- Other (please specify)………

1. Please indicate the MINIMUM level of HYPEROPIA (in dioptric value) at which you would consider prescribing spectacles in a NON-STRABISMIC child of the following ages:

|  | Less than 1D | 1-2 D | 2-3 D | 3-4 D | More than 4 D |
| --- | --- | --- | --- | --- | --- |
| 1 year |  |  |  |  |  |
| 3 years |  |  |  |  |  |
| 5 years |  |  |  |  |  |
| 7 years |  |  |  |  |  |
| 9 years |  |  |  |  |  |
| 11 years |  |  |  |  |  |

1. Please indicate the MINIMUM level of MYOPIA (in dioptric value) at which you would consider prescribing spectacles in a NON-STRABISMIC child of the following ages:

|  | Less than 1D | 1-2 D | 2-3 D | 3-4 D | More than 4 D |
| --- | --- | --- | --- | --- | --- |
| 1 year |  |  |  |  |  |
| 3 years |  |  |  |  |  |
| 5 years |  |  |  |  |  |
| 7 years |  |  |  |  |  |
| 9 years |  |  |  |  |  |
| 11 years |  |  |  |  |  |

1. Please indicate the MINIMUM level of ANISOMETROPIA (in dioptric value) at which you would consider prescribing spectacles in a NON-STRABISMIC child of the following ages:

|  | Less than 1D | 1-2 D | 2-3 D | 3-4 D | More than 4 D |
| --- | --- | --- | --- | --- | --- |
| 1 year |  |  |  |  |  |
| 3 years |  |  |  |  |  |
| 5 years |  |  |  |  |  |
| 7 years |  |  |  |  |  |
| 9 years |  |  |  |  |  |
| 11 years |  |  |  |  |  |

1. Please indicate the MINIMUM level of NON-OBLIQUE ASTIGMATISM at which you would consider prescribing spectacles in a NON-STRABISMIC child of the following ages:

|  | Less than 1D | 1-2 D | 2-3 D | 3-4 D | More than 4 D |
| --- | --- | --- | --- | --- | --- |
| 1 year |  |  |  |  |  |
| 3 years |  |  |  |  |  |
| 5 years |  |  |  |  |  |
| 7 years |  |  |  |  |  |
| 9 years |  |  |  |  |  |
| 11 years |  |  |  |  |  |

1. Please indicate the MINIMUM level of OBLIQUE ASTIGMATISM at which you would consider prescribing spectacles in a NON-STRABISMIC child of the following ages:

|  | Less than 1D | 1-2 D | 2-3 D | 3-4 D | More than 4 D |
| --- | --- | --- | --- | --- | --- |
| 1 year |  |  |  |  |  |
| 3 years |  |  |  |  |  |
| 5 years |  |  |  |  |  |
| 7 years |  |  |  |  |  |
| 9 years |  |  |  |  |  |
| 11 years |  |  |  |  |  |

1. What factors influence whether or not you prescribe for bilateral hyperopia? (multiple selection you may choose more than one choice)

- Symptoms
- Presence of astigmatism/ anisometropia
- Reading problems
- Accommodative dysfunction
- Esophoria
- Presence of motor/ neurodevelopmental problems
- Decrease near visual acuity
- Decrease stereoacuity
- Refractive error at prior eye exam
- Family history
- Parent or child preferences

1. I prescribe correction for bilateral hyperopia if present with **symptoms** at:

- 1 Diopter
- 2 Diopters
- 3 Diopters
- 4 Diopters
- Not prescribe

1. I prescribe correction for bilateral hyperopia if present with **reading problems** at:

- 1 Diopter
- 2 Diopters
- 3 Diopters
- 4 Diopters
- Not prescribe

1. prescribe correction for bilateral hyperopia if present with **accommodative dysfunction** at:

- 1 Diopter
- 2 Diopters
- 3 Diopters
- 4 Diopters
- Not prescribe

1. I prescribe correction for bilateral hyperopia if present with **esophoria** at:

- 1 Diopter
- 2 Diopters
- 3 Diopters
- 4 Diopters
- Not prescribe

1. I prescribe correction for bilateral hyperopia if present with **motor/neurodevelopmental problems** at:

- 1 Diopter
- 2 Diopters
- 3 Diopters
- 4 Diopters
- Not prescribe

1. I prescribe correction for bilateral hyperopia if present with **decrease near visual acuity** at:

- 1 Diopter
- 2 Diopters
- 3 Diopters
- 4 Diopters
- Not prescribe

1. I prescribe correction for bilateral hyperopia if present with **decrease stereoacuity** at:

- 1 Diopter
- 2 Diopters
- 3 Diopters
- 4 Diopters
- Not prescribe

1. I prescribe correction for bilateral hyperopia if present with **refractive error at prior eye exam** at:

- 1 Diopter
- 2 Diopters
- 3 Diopters
- 4 Diopters
- Not prescribe

1. I prescribe correction for bilateral hyperopia if present with **family history** at:

- 1 Diopter
- 2 Diopters
- 3 Diopters
- 4 Diopters
- Not prescribe

1. I prescribe correction for bilateral hyperopia if present with **parent or child preferences** at:

- 1 Diopter
- 2 Diopters
- 3 Diopters
- 4 Diopters
- Not prescribe

1. In your opinion what best explains your prescribing pattern of hyperopia

- Experience and clinical opinion judgment
- Guideline of American Association of Pediatric Ophthalmology and Strabismus
- Guideline of American Academy of Ophthalmology
- Guideline of, Royal College of Ophthalmologists.
- Guideline suggested by Susan Leat (2011)
